# Gazefinder as a clinical supplementary tool for discriminating between autism spectrum disorder and typical development in male adolescents and adults

**DOI:** 10.1186/s13229-016-0083-y

**Published:** 2016-03-23

**Authors:** Toru Fujioka, Keisuke Inohara, Yuko Okamoto, Yasuhiro Masuya, Makoto Ishitobi, Daisuke N. Saito, Minyoung Jung, Sumiyoshi Arai, Yukiko Matsumura, Takashi X. Fujisawa, Kosuke Narita, Katsuaki Suzuki, Kenji J. Tsuchiya, Norio Mori, Taiichi Katayama, Makoto Sato, Toshio Munesue, Hidehiko Okazawa, Akemi Tomoda, Yuji Wada, Hirotaka Kosaka

**Affiliations:** Department of Neuropsychiatry, Faculty of Medical Sciences, University of Fukui, Eiheiji, Fukui 910-1193 Japan; Research Center for Child Mental Development, University of Fukui, Eiheiji, Fukui 910-1193 Japan; Department of Child Development, United Graduate School of Child Development, Osaka University, Kanazawa University, Hamamatsu University School of Medicine, Chiba University, and University of Fukui, Suita, Osaka 565-0871 Japan; Department of Informatics, Graduate School of Informatics and Engineering, The University of Electro-Communications, Chofu, Tokyo, 182-8585 Japan; Department of Child and Adolescent Mental Health, National Institute of Mental Health, National Center of Neurology and Psychiatry, Kodaira, Tokyo, 187-8553 Japan; Biomedical Imaging Research Center, University of Fukui, Eiheiji, Fukui 910-1193 Japan; Department of Psychiatry and Human Behavior, Gunma University Graduate School of Medicine, Maebashi, Gunma 371-8511 Japan; Research Center for Child Mental Development, Hamamatsu University School of Medicine, Hamamatsu, Shizuoka 431-3192 Japan; Department of Psychiatry, Hamamatsu University School of Medicine, Hamamatsu, Shizuoka 431-3192 Japan; Research Center for Children’s Mental Development, United Graduate School of Child Development, Suita, Osaka 565-0871 Japan; Department of Anatomy and Neuroscience, Graduate School of Medicine, Osaka University, Suita, Osaka 565-0871 Japan; Research Center for Child Mental Development, Kanazawa University, Kanazawa, Ishikawa 920-8641 Japan

**Keywords:** Gaze abnormality, Autism spectrum disorder, Eye-tracking, Face, Biological motion, Geometry, Discriminant analysis, Fixation, Adult, Adolescent

## Abstract

**Background:**

Gaze abnormality is a diagnostic criterion for autism spectrum disorder (ASD). However, few easy-to-use clinical tools exist to evaluate the unique eye-gaze patterns of ASD. Recently, we developed Gazefinder, an all-in-one eye-tracking system for early detection of ASD in toddlers. Because abnormal gaze patterns have been documented in various ASD age groups, we predicted that Gazefinder might also detect gaze abnormality in adolescents and adults. In this study, we tested whether Gazefinder could identify unique gaze patterns in adolescents and adults with ASD.

**Methods:**

We measured the percentage of eye fixation time allocated to particular objects depicted in movies (i.e., eyes and mouth in human face movies, upright and inverted biological motion in movies that presented these stimuli simultaneously, and people and geometry in movies that presented these stimuli simultaneously) by male adolescents and adults with ASD (*N* = 26) and age-matched males with typical development (TD; *N* = 35). We compared these percentages between the two groups (ASD and TD) and with scores on the social responsiveness scale (SRS). Further, we conducted discriminant analyses to determine if fixation times allocated to particular objects could be used to discriminate between individuals with and without ASD.

**Results:**

Compared with the TD group, the ASD group showed significantly less fixation time at locations of salient social information (i.e., eyes in the movie of human faces without lip movement and people in the movie of people and geometry), while there were no significant groupwise differences in the responses to movies of human faces with lip movement or biological motion. In a within-group correlation analysis, a few of the fixation-time items correlated with SRS, although most of them did not. No items significantly correlated with SRS in both ASD and TD groups. The percentage fixation times to eyes and people, which exhibited large effect sizes for the group difference, could differentiate ASD and TD with a sensitivity of 81.0 % and a specificity of 80.0 %.

**Conclusions:**

These findings suggest that Gazefinder is potentially a valuable and easy-to-use tool for objectively measuring unique gaze patterns and discriminating between ASD and TD in male adolescents and adults.

**Electronic supplementary material:**

The online version of this article (doi:10.1186/s13229-016-0083-y) contains supplementary material, which is available to authorized users.

## Background

Autism spectrum disorder (ASD) is a neurodevelopmental condition characterized by “deficits in social communication and social interaction” and “restricted, repetitive patterns of behavior, interests, or activities,” according to the fifth edition of the *Diagnostic and Statistical Manual of Mental Disorders* (DSM-5) [1]. The phrase “never looked into anyone’s face” was used in the first report about autism [2], and this unique gaze fixation pattern is a characteristic included in the diagnostic criteria for ASD [1]. However, because a medical staff subjectively assesses gaze abnormality through clinical examination of patients or interview with caregivers, documentation of the severity of a patient’s gaze abnormality can differ among medical evaluators. To more objectively measure gaze abnormalities in individuals with ASD, it is important that clinicians have access to easy-to-use clinical tools.

A number of studies have identified unique visual gaze patterns in individuals with ASD using eye-tracking systems, for example, Tobii® (Tobii Technology; Stockholm, Sweden) or ISCAN® (ISCAN Inc.; Woburn, MA, USA) [3–19]. Using these systems, gaze abnormalities in individuals with ASD were detected when they observed movies or photographs of human faces [3–12], biological motion [13–16], people [3, 17–23], and the simultaneous presentation of people and geometric stimuli [24, 25]. For instance, compared with age-matched typically developed (TD) individuals, children [3–6, 10, 11, 20–23] and adults [7–9, 12, 19, 26] with ASD spent less time gazing at eye or face regions. These abnormalities in individuals with ASD were consistently observed in various types of tasks, such as free-viewing tasks [3, 5, 6, 9, 10, 19–23], facial emotion discrimination tasks [4, 9], and facial recognition tasks [4, 7, 8, 11]. In addition, several studies have reported abnormal gaze when children with ASD view biological motion [13–16]. Three of these studies used simultaneous presentation of upright and inverted biological motion [13, 15, 16], and two studies revealed that children with ASD gaze less at the upright biological motion. On the other hand, Fujisawa et al. [15] showed that preschool children with ASD exhibited stronger preference for upright biological motion as compared with age-matched TD children. These findings suggest that the abnormality in the preference for upright biological motion in individuals with ASD might change with age and serve to diagnose ASD. Furthermore, previous studies revealed that children with ASD preferred looking at geometric shapes when people and geometric shapes were presented simultaneously [24, 25]. The report by Shi et al. [25] showed high sensitivity and specificity using total fixation time with images of people, namely 84.6 and 85.0 %, respectively. These findings imply that fixation time on particular classes of objects as measured by eye-tracking systems might provide an objective assessment of gaze abnormalities in both children and adults with ASD.

Such gaze fixation patterns in individuals with ASD are considered to be associated with particular symptoms (e.g., social deficits). For instance, the social affect score of the autism diagnostic observation schedule (ADOS) [27] correlated negatively with the fixation time to human faces or eyes in toddlers with ASD [5, 18]. Additionally, scores on the social responsiveness scale (SRS) [28] were negatively correlated with the fixation time to human eyes in groups including both ASD and TD children [6]. The findings imply that fixation time to a particular object as measured by eye-tracking systems could be used as a predictor of social-deficit severity for each individual.

Although these eye-tracking techniques can objectively evaluate the gaze pattern of individuals with ASD, it is necessary for scientists conducting basic research on ASD-related gaze to develop original and experimental approaches and stimuli expressly designed to discover and explore the unique characteristics of abnormal gaze. However, this design process is not practical in clinical settings because of limited work force and/or time. To address the clinical need for an easy-to-use system, the Gazefinder (JVC KENWOOD Corporation, Kanagawa, Japan), a simple all-in-one eye-tracking system, has been developed for early detection of ASD in toddlers. Gazefinder incorporates movies of human faces, biological motion, and people and geometric shapes, and it provides almost instantaneous data by automatically calculating the percentage fixation time allocated by the participants among regions of interest in the movie. Because Gazefinder was designed for toddlers, the total trial time is short (approximately 2 min), and instructions or verbal answers are not required; the participant simply views a video monitor. We reported that, compared with TD children, preschool children with ASD exhibited a more prominent preference for (i.e., higher percentage of visual attention to) upright biological motion over inverted biological motion when assessed with Gazefinder [15]. Therefore, the percentage fixation time to biological motion, as determined by Gazefinder, could be used for evaluating gaze abnormality in ASD preschool children. Given that gaze abnormality has been found not only in children [3–6, 13–15, 17–19] but also in adults with ASD [7–9, 19], it is possible that Gazefinder could be used for evaluating the gaze patterns of adolescents and adults with ASD. Brugha and colleagues reported that the estimated prevalence of ASD in an adult population was approximately one percent, but all cases detected in this research were not diagnosed [29]. Considering that the concept of adult ASD is gaining increasing acceptance, objective assessments in addition to traditional subjective assessments (e.g., listening or questionnaires) will be helpful in detecting ASD traits that might otherwise go undiagnosed, and Gazefinder could be useful here. However, because it has been discovered that the gaze fixation pattern of adults with ASD is different from that of children with ASD [19], the gaze fixation patterns measured by Gazefinder should also reveal changes with age. No studies to date have examined gaze patterns in adolescents and adults with ASD using Gazefinder. Therefore, before using Gazefinder with the adolescent-to-adult age group in the clinical setting, it is necessary to examine whether it can detect the specific characteristics of ASD gaze patterns in this age group. In addition, it is also necessary to affirm whether gaze fixation patterns measured by Gazefinder reflect sociality and distinguish individuals with ASD from TD individuals in this age group.

Using Gazefinder, we first examined the gaze characteristics of adolescents and adults with ASD. Specifically, we examined (1) the percentage fixation time allocated to specific objects in individuals with and without ASD and (2) the relationships between the percentage fixation time and the severity of social deficit. We then examined (3) if individuals with and without ASD can be distinguished from each other by the percentage fixation times.

## Methods

### Participants

We recruited 26 male adolescents and adults with ASD and 35 age-matched TD male participants. The protocol used for this study was approved by the ethics committee of the University of Fukui. After a complete explanation of the study, all participants or their parents/legal guardians provided written, informed consent. We obtained SRS scores [30] to determine the severity of social deficit exhibited by participants with ASD and TD. In the present study, we used the 65-item SRS adult self-report form. Each item is scored on the Likert scale, which ranges from 0 (never true) to 3 (almost always true). Total SRS scores range from 0 to 195, with higher total scores indicating more severe social deficits.

### The ASD group

A total of 26 male adolescents and adults with ASD (15 to 41 years old) were recruited by the Department of Neuropsychiatry at the University of Fukui Hospital, Japan, and the Department of Psychiatry and Neurobiology at the Kanazawa University Hospital, Japan. Two authors (TM and HK) diagnosed the participants based on the criteria of the DSM-IV-TR [31] and on the standardized criteria of the Diagnostic Interview for Social and Communication Disorders (DISCO) [32]. The latter has good psychometric properties [33]. DISCO also contains items concerning early development and a section on activities of daily living that provide data regarding functioning in areas other than in the social and communication domains [32]. Participants were excluded if they met the diagnostic criteria for any other psychopathology, e.g., attention-deficit/hyperactivity disorder (ADHD), schizophrenia spectrum, anxiety disorder, or obsessive-compulsive disorder. A total of 23 individuals with ASD completed the Wechsler Adult Intelligence Scale—Third Edition (WAIS-III) [34], and one participant who was 15 years old completed the Wechsler Intelligence Scale for Children—Third Edition (WISC-III) [35], for assessment of intelligence quotient (IQ). All participants scored 70 or higher on the full-scale IQ (FSIQ). The IQs of two individuals with ASD who refused WAIS-III were recognized to be average based on their past or current academic performance.

### The TD group

We also recruited 35 age-matched TD male participants (20 to 41 years old) from the local community. Individuals with a history of major medical or neurological illness including epilepsy, significant head trauma, or a lifetime history of alcohol or drug dependence were excluded from this study. We performed screenings to exclude individuals who had a first-degree relative with an Axis I disorder diagnosed by DSM-IV-TR criteria [31].

### Eye-tracking system with Gazefinder

**Fig. 1:**
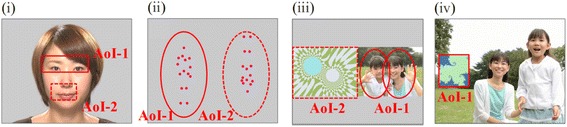
Gazefinder movie samples. **i** Human face; AoI-1 and AoI-2 include the eye and mouth regions, respectively. **ii** Biological motion; AoI-1 and AoI-2 are the upright and inverted images, respectively. **iii** People and geometry (same size); AoI-1 and AoI-2 are same-sized images of people and geometry, respectively. **iv** People and geometry (small window). AoI-1, the geometric image; AoI, area-of-interest. We had permission to use the samples of Gazefinder presented in Fig. 1 from JVC KENWOOD Corporation

We utilized Gazefinder, an all-in-one eye-tracking system, to evaluate the percentage fixation times allocated to specific objects (see Fig. 1) on a video monitor. The experiment was conducted in a quiet laboratory at the University of Fukui.

### Measurement of eye position

Participants’ eye position was measured using infrared light sources and cameras located below a 19-inch thin-film transistor (1280 × 1024 pixels). Using corneal reflection techniques, eye position was recorded as *X* and *Y* coordinates at a frequency of 50 Hz (i.e., 3000 data collections/min). Calibration of the eye position recording was performed with a five-point method, whereby each participant followed the location of a ball around the screen before a series of movies were presented.

### Stimuli

After calibration, eight short movies were presented, including five movies of a human face, a movie of biological motion, and two movies of people and geometry. Human face movie clips consisted of the following: (A) still image (7 s), (B) eye blinking (an actress repeatedly opens and closes her eyes for 7 s), (C) mouth moving (an actress repeatedly opens and closes her mouth for 4 s), (D) silent (an actress with a still face for 3 s), and (E) talking (7 s) (Fig. 1i). In (E), the actress says, “*Konnichiwa*” (“Hello” in English), “*Onamaewa*?” (“What is your name?” in English), and “*Issyoniasobouyo*” (“Let’s play together” in English). Movie (F) presents upright and inverted biological motion simultaneously (20 s) (Fig. 1ii). This movie was accompanied by a soundtrack playing the song *Under the Big Chestnut Tree*, to which an upright human (i.e., biological motion) danced. Similarly, an inverted human (i.e., upside-down biological motion) was played backwards, relative to the movie of the upright biological motion. Movies of people and geometry consisted of (G) people and geometry presented at the same time and at the same size for 16 s and (H) a movie of geometry depicted in small-frame images in a small window on a movie of people, for 16 s (Fig. 1iii, iv). The movies were sequentially displayed, beginning with the human face (still image) (A) all the way through to the movies of people and geometry (small window) (H). Animations to reorient the viewer’s attention to the stimuli (e.g., animation with a voice saying “Hey! Look!”) were presented on the screen after (B) human face (blinking), (E) human face (talking), (F) biological motion, and (G) people and geometry (same size) and at the midpoint of (G) people and geometry (same size). The rationale for the design of these Gazefinder stimuli is given in Additional file 1.

### Analysis of the percentage fixation times

Percentage fixation times allocated to particular areas on the video monitor were automatically calculated (time allocated to a particular area/duration of stimulus presentation), and the results could be printed immediately after the videos were viewed. Figure 1 shows areas-of-interest (AoIs) for each stimulus. Human face included two AoIs (i.e., AoI-1 is the eye region and AoI-2 is the mouth region) (Fig. 1i). The biological motion includes two AoIs (i.e., AoI-1 is upright biological motion involving dancing to the song, and AoI-2 is inverted biological motion that was presented upside down and played backwards relative to the upright biological motion) (Fig. 1ii). For the movie of people and geometry (same size), there were two AoIs (i.e., AoI-1 is the area with people, and AoI-2 is the area with geometry) (Fig. 1iii). The people and geometry (small window) movie contained one AoI (i.e., the geometric shapes window) (Fig. 1iv). In addition to these AoIs, the percentage fixation time allocated to areas other than AoI(s) (i.e., out of AoI) was also calculated during the movies of the human faces and people and geometry.

### Analysis of data

#### Exclusion criteria

Individuals for whom the available percentage fixation time was <80 % were excluded (i.e., Gazefinder could not detect eye position more than 20 % of the time).

### Statistical analysis

We first investigated differences in gaze patterns between the ASD and TD groups. Although we could conduct ANOVA with face stimuli and biological motion, we could not carry out ANOVA that included movies of people and geometry due to a mismatch in the number of AoIs. Therefore, in order to unify statistical methods and to clarify group differences in the effect of each AoI of a stimulus, we conducted independent *t* tests for percentage fixation times to each AoI. To avoid type 1 statistical errors, we applied Bonferroni corrections and set .05 divided by the number of AoIs as the significance level; we set .025 (.05/2) as the significance level for the stimuli that included human faces, biological motion, and people and geometry (same size). As was the case with the movies having only the AoI-1, we set .05 as the statistically significant level in the people and geometry (small window) condition. We also calculated effect size (i.e., Cohen’s *d*) for the differences between the ASD and TD groups, setting *d* > 0.5 as medium and *d* > 0.8 as large, in accordance with the criteria of Cohen [36]. We then conducted a correlation analysis. Because the unique gaze pattern of ASD may reflect social deficits selectively [18], we examined whether the percentage fixation times correlated with raw scores on the SRS, which measures the social deficits characteristic of ASD. In addition to this, we also examined the correlation between the percentage fixation times and the FSIQ, to test whether intellectual ability affected group differences or correlations. Finally, we investigated whether the percentage fixation times to AoI discriminate between ASD and TD. More specifically, we conducted receiver operating characteristic (ROC) analysis and calculated the area under the curves (AUCs) to establish cutoff points that would serve as thresholds indicating an ASD-like pattern for each AoI. Incidentally, AUC reflects the discriminant level in ROC analysis, and ≥.7 is considered acceptable and ≥.8 is considered excellent [37]. Based on these results, we initially selected all AoI-1s shown in Fig. 1 and evaluated sensitivity, specificity, positive likelihood ratio (PLR), and negative likelihood ratio (NLR), broken down by the number of items above the cutoff point. Then, because items that did not show a clear group difference were thought to decrease the accuracy of the discriminant parameters (i.e., sensitivity, specificity, PLR, and NLR), we evaluated these parameters excluding the item that could not discriminate well between the ASD and TD groups. In particular, we found AoI-1s that showed the large effect size (*d* > 0.8) and calculated the discriminant parameters according to the number of items that met the cutoff condition. All statistical analyses were conducted using IBM SPSS statistical software, version 22.

## Results

### Demographic data of participants used in data analysis

Five participants in the ASD group for whom the available percentage fixation times were <80 % were excluded from the study (the available percentage fixation times of the excluded participants were 60, 58, 53, 52, and 31 %). There were no excluded individuals in the TD group. Thus, data for 21 individuals with ASD and 35 TD individuals were used for the following analysis. All 21 participants with ASD completed the WAIS-III or WISC-III. After excluding the five participants with ASD, the demographic data were as presented in Table 1. There was no significant group difference in age, and the ASD group showed significantly higher SRS scores (Table 1).

**Table 1:** Age, IQ, and SRS scores of participants

	ASD (*n =* 21)	TD (*n =* 35)	*t* value	*p*
Age (years)	27.6 ± 7.7	25.2 ± 4.5	1.28	.212
WAIS-III^a^				
Full-scale IQ	99.8 ± 13.5			
Verbal IQ	103.3 ± 13.3			
Performance IQ	96.4 ± 16.0			
SRS^b^	111.8 ± 18.5	53.6 ± 16.9	11.46	<.001

*ASD* autism spectrum disorders, *IQ* intelligent quotient, *SRS* social responsiveness scale, *TD* typically developed, *WAIS-III* Wechsler Adult Intelligence Scale—Third Edition, *WISC-III* Wechsler Intelligence Scale for Children—Third Edition

^a^ *n* = 20 (one excluded participant that completed WISC-III; full-scale IQ = 115)

^b^ *n* = 52 (four data points are missing in the ASD group)

### Comparing the percentage fixation times (ASD vs. TD)

Preliminary independent *t* test found no group difference in the available percentage fixation times between the ASD and TD groups (Table 2). We then compared the percentage fixation times devoted to each AoI (time allocated to each AoI/duration of each stimulus presentation) between the two groups (ASD and TD).

**Table 2:** Mean fixation percentages and group differences of each item

	ASD (*n* = 21)	TD (*n* = 35)	*t* value	*p*	Effect size Cohen’s *d*
The available percentage fixation	95.3 ± 5.5	97.2 ± 3.1	1.45	.157	*d* = 0.47
Human face					
A) Still image					
% eyes	64.9 ± 22.9	80.9 ± 19.0	2.83	.007*	*d* = 0.79^a^
% mouth	11.5 ± 10.1	7.5 ± 8.3	1.59	.118	*d* = 0.45
B) Blinking					
% eyes	46.7 ± 31.6	77.4 ± 24.1	3.83	.001*	*d* = 1.15^b^
% mouth	22.1 ± 19.2	7.0 ± 15.7	3.02	.005*	*d* =0.89^b^
C) Mouth moving					
% eyes	36.3 ± 27.3	49.3 ± 21.7	1.96	.055	*d* = 0.55^a^
% mouth	36.2 ± 25.7	27.7 ± 20.5	1.36	.179	*d* = 0.38
D) Silent					
% eyes	28.9 ± 36.4	60.0 ± 31.0	3.40	.001*	*d* = 0.96^b^
% mouth	38.2 ± 34.6	17.7 ± 22.3	2.43	.021*	*d* = 0.76^a^
E) Talking					
% eyes	37.1 ± 34.7	49.4 ± 27.3	1.48	.145	*d* = 0.42
% mouth	36.8 ± 32.4	28.3 ± 21.8	1.06	.298	*d* = 0.33
Biological motion					
F) Biological motion					
% upright	47.7 ± 13.4	52.7 ± 16.3	1.18	.243	*d* = 0.33
% inverted	49.7 ± 13.8	44.7 ± 15.6	1.21	.232	*d* = 0.34
People and geometry					
G) Same size					
% people	47.6 ± 23.1	67.2 ± 16.6	3.39	.002*	*d* = 1.03^b^
% geometry	44.7 ± 22.3	29.8 ± 15.9	2.67	.012*	*d* = 0.82^b^
H) Small window					
% geometry	33.0 ± 23.9	21.9 ± 15.3	2.13	.038**	*d* = 0.60^a^

*p < .025 (.05/2); **p < .050

^a^Moderate effect size (>0.50)

^b^High effect size (>0.80) (Cohen, [36])

**Fig. 2:**
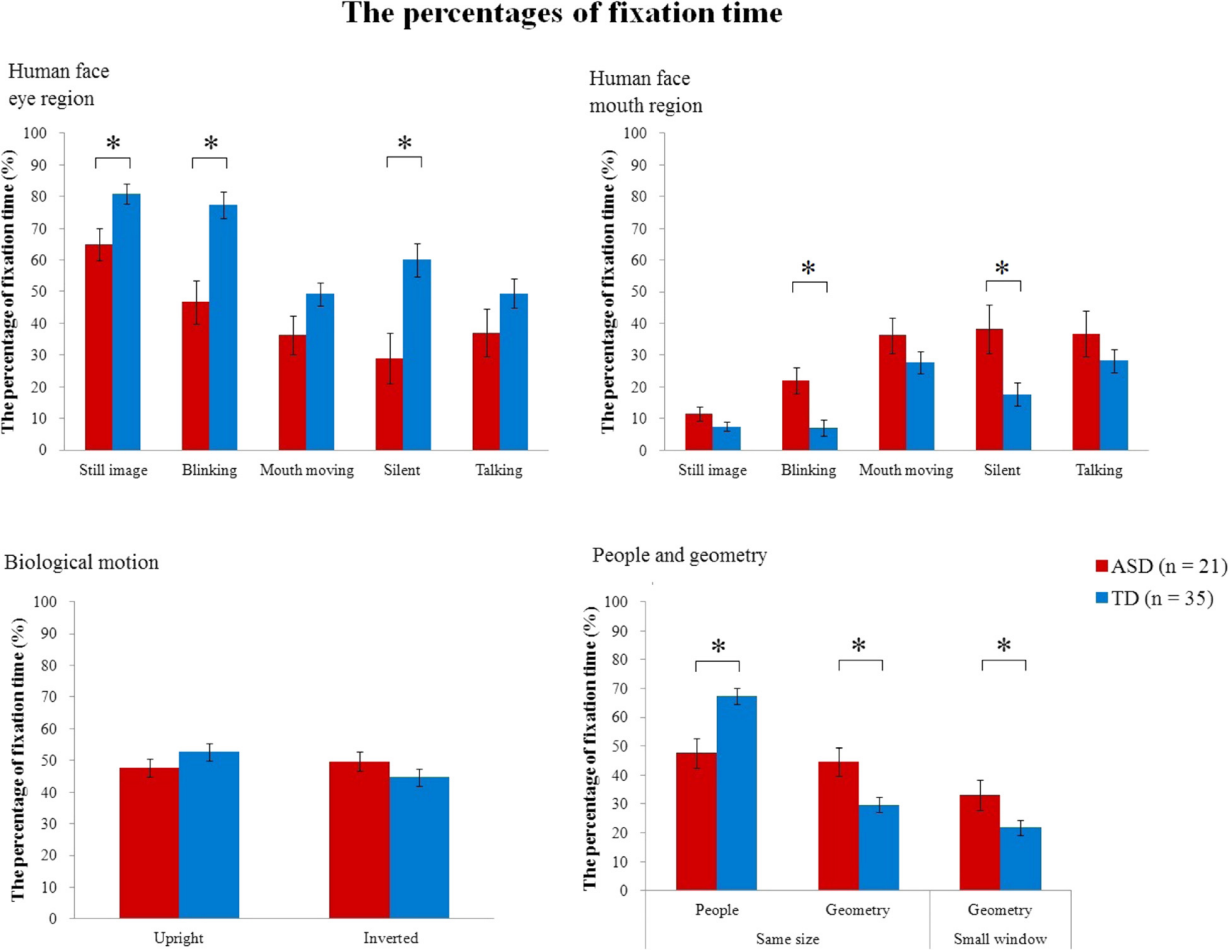
Bar graphs of the percentage fixation times and standard errors of each AoI. *Below significance level after Bonferroni correction; .025 (.05/2) for the movies of human faces, biological motion, and people and geometry (same size) and .05 for the people and geometry (small window). AoI, area-of-interest

For human face stimuli, the percentage fixation times to the eyes (AoI-1) in (still image), (blinking), and (silent) were significantly lower in the ASD group than those in the TD group, and the percentage fixation times to the mouth were higher in the ASD group than those in the TD group for (blinking) and (silent) (all after Bonferroni corrections). For people and geometry (same size), the percentage fixation times allocated by the ASD group were lower for people (AoI-1) and higher for geometry than the corresponding values in the TD group, after Bonferroni corrections. For people and geometry (small window), the percentage fixation time to geometry (AoI-1) was higher in the ASD group than that in the TD group. We could not find significant group differences in other face stimuli (Table 2 and Fig. 2).

### Correlation analysis with social response scale

Because significant group differences were found in the percentage fixation times described above, correlations in whole-group analysis reflect group differences instead of real continuous dimensions. Therefore, we analyzed the within-group correlations between the percentage fixation on each AoI and SRS score to determine whether gaze abnormality reflects social deficits in each group. We found only two significant correlations; one was a positive correlation for time devoted to the mouth region in the face (blinking) in the ASD group, and the other was a positive correlation for the geometry region of people and geometry stimuli in the TD group. Other items were not significantly correlated (Table 3).

**Table 3:** Correlations between the percentage fixation times and psychometric test scores

	SRS^a^				FSIQ^b^	
	ASD group		TD group			
	*r*	*p*	*r*	*p*	*r*	*p*
Human face						
A) Still image						
% eyes	−.31	.233	.09	.613	−.42	.058
% mouth	.19	.461	−.06	.738	.17	.457
B) Blinking						
% eyes	−.41	.104	.09	.594	−.15	.511
% mouth	.50*	.037	−.02	.909	.10	.665
C) Mouth moving						
% eyes	−.17	.508	.07	.683	−.31	.169
% mouth	.31	.226	.08	.665	.33	.146
D) Silent						
% eyes	−.21	.410	−.01	.957	−.27	.237
% mouth	.44	.080	−.01	.957	.31	.166
E) Talking						
% eyes	−.20	.433	−.10	.593	−.38	.092
% mouth	.25	.333	.25	.147	.32	.154
Biological motion						
F) Biological motion						
% upright	−.46	.066	.26	.132	−.12	.617
% inverted	.35	.170	−.26	.144	.10	.680
People and geometry						
G) Same size						
% people	−.01	.971	−.33	.057	−.25	.284
% geometry	.04	.869	.39*	.022	.20	.382
H) Small window						
% geometry	.17	.511	.14	.427	−.08	.737

*ASD* autism spectrum disorders, *TD* typically developed, *SRS* social responsiveness scale, *FSIQ* full-scale intelligence quotient (WAIS-III)

*p < .05

^a^ *n* = 52 (ASD = 17, TD = 35)

^b^ASD group only (*n* = 21)

We also evaluated the correlations between the percentage fixation times and the FSIQ, in order to confirm if intellectual ability affected any of the group differences or correlations described above. Although FSIQ scores were only measured in the ASD group, FSIQ scores were not found to correlate with any fixation percentages (Table 3).

### Discriminant analysis

Finally, we examined whether the percentage fixation time could differentiate between ASD and TD. After conducting an ROC analysis to define the cutoff point/threshold for each AoI, we investigated whether we could distinguish participants with ASD from TD participants using all the AoI-1s. Next, because items that did not reveal a clear group difference were thought to reduce discrimination power, we examined whether we could distinguish participants with ASD from TD participants by using only AoI-1s that had large effect sizes in the group differences for the percentage fixation times.

### ROC analysis in each AoI

The AUCs and threshold/cutoff point for each AoI are shown in Table 4. For human faces, the threshold for each AoI-1 (i.e., eye region) was <71 % (still image), <81 % (blinking), <20 % (mouth moving), <32 % (silent), and <11 % (talking). For biological motion, the threshold of each AoI-1 (upright) was <50 %, and those of people and geometry were <57 % for the people region in (same size) and >31 % for the geometric region in (small window) (Table 4).

**Table 4:** Area under the curve and cutoff points for each item

	AUC	95 % CI	Cutoff point (%)
Human face			
A) Still image			
% eyes	.73	.60–.87	<71
% mouth	.63	.48–.78	>7
B) Blinking			
% eyes	.80	.68–.92	<81
% mouth	.79	.65–.92	>8
C) Mouth moving			
% eyes	.65	.50–.81	<20
% mouth	.59	.43–.75	>32
D) Silent			
% eyes	.75	.60–.89	<32
% mouth	.66	.50–.82	>16
E) Talking			
% eyes	.62	.46–.80	<11
% mouth	.54	.38–.71	>38
Biological motion			
F) Biological motion			
% upright	.59	.44–.75	<50
% inverted	.59	.43–.75	>48
People and geometry			
G) Same size			
% people	.74	.60–.88	<57
% geometry	.69	.54–.84	>37
H) Small window			
% geometry	.62	.46–.78	>31

*AUC* area under the curve, *CI* confidence interval

### Discriminant analysis using all AoI-1s

Using all the AoI-1s from all the eight stimuli (Fig. 1), we counted the number of items that met the cutoff condition defined by the ROC analysis for each participant. The highest discriminant parameters were found when we defined participants who had more than four items that met the cutoff condition as “ASD.” The sensitivity, specificity, PLR, and NLR were 61.9 %, 85.7 %, 4.3, and 0.4, respectively.

### Discriminant analysis using AoI-1s having the largest effect size

**Fig. 3:**
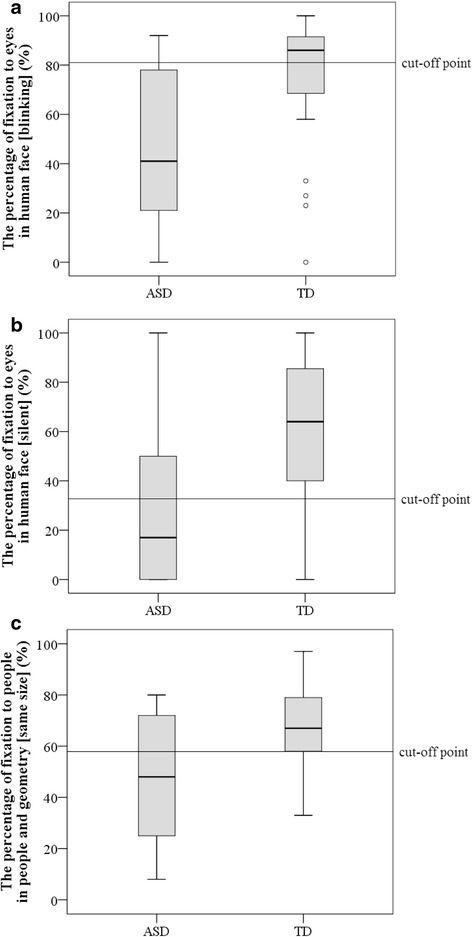
Boxplots of stimuli used for discriminant analysis. **a** The percentage of visual fixation allocated to the eyes in the human face (blinking). **b** The percentage fixation to the eyes in the human face (silent). **c** The percentage fixation to people in people and geometry (same size)

We then examined discrimination parameters utilizing the AoI-1s that had revealed the largest effect size (*d* > 0.8), which included the eye region in the human face (blinking) and (silent), and the people region in people and geometry (same size). Within these three AoIs, we counted the number of items that met the cutoff condition defined by the ROC analysis for each participant (see Fig. 3 and Table 5). When we defined participants who had more than two items meeting the cutoff condition as “ASD,” we found that the highest discrimination parameters included sensitivity, specificity, PLR, and NLR, which were 81.0 %, 80.0 %, 4.1, and 0.2, respectively (Table 6).

**Table 5:** Breakdown of number of items above cutoff

	ASD		TD	
	*n*	%	*n*	%
No stimulus over cutoff				
None of the three stimuli	1	4.8	18	51.4
One stimulus over cutoff				
Human face (blinking) only	2	9.5	5	14.3
Human face (silent) only	0	0.0	2	5.7
People and geometry (same size) only	1	4.8	3	8.6
Two stimuli over cutoff				
Human face (blinking) and human face (silent) only	5	23.8	3	8.6
Human face (blinking) and people and geometry (same size) only	2	9.5	0	0.0
Human face (silent) and people and geometry (same size) only	1	4.8	1	2.9
Three stimuli over cutoff				
All three stimuli	9	42.9	3	8.6
Total	21	100.0	35	100.0

% is the percentage of the number of participants for each group

*ASD* autism spectrum disorders, *TD* typically developed

**Table 6:** Parameters of discriminant analysis

The number of items over cutoff point	Sensitivity (%)	Specificity (%)	PLR	NLR
>1	95.2	51.4	2.0	0.1
>2	81.0	80.0	4.1	0.2
>3	42.9	91.4	5.0	0.6

This discriminant analysis selected the percentage fixation times to (1) eyes in human faces in (blinking), (2) eyes in human faces in (silent), and (3) people in people and geometry (same size). Cutoff points were <81, <32, and <57, respectively

*PLR* positive likelihood ratio, *NLR* negative likelihood ratio

## Discussion

### Group differences in percentage fixation times and relationship between percentage fixation times and social disability

In the present study, we found significant group differences in the percentage fixation time allocated to areas within movies of human faces (still image, blinking, and silent) and movies showing people and geometry, while we could not find significant group differences in other movies of human faces (mouth moving and talking), or in the movie of biological motion. Within-group correlation analysis found a few significant correlations between the percentage fixation times and SRS, although most of them did not show significant correlations and there were no items significantly correlated in both the ASD and TD groups.

### Group differences in percentage fixation times

The ASD group gazed less at eyes in human face stimuli (still image), (blinking), and (silent), while we could not find a group difference for the eyes in human face (mouth moving) and (talking). One explanation is that the movement of the lips included in the human face (mouth moving) and (talking) reduce attention to the eyes in the TD group, which in turn reduced group differences in fixation time to the eye region. Actually, although we did not perform a statistical comparison among stimulus conditions, our results showed that the fixation percentages of the TD group for the eye region in human faces in (still image), (blinking), and (silent) stimuli were relatively high (80.9, 77.4, and 60.0, respectively), while those in the eye region in human faces in (mouth moving) and (talking) stimuli were relatively low (49.4 and 49.3, respectively). Therefore, our results suggest that the abnormal gaze pattern in individuals with ASD (i.e., reduced attention to the eye and increased attention to the mouth) becomes less prominent when these participants view face stimuli that include movement of the lips. This interpretation is consistent with the report by Chawarska et al. [20], suggesting that toddlers with ASD tend to gaze at moving objects.

For biological motion, our data did not show different gaze patterns between the ASD and TD groups, unlike previous studies of children with ASD [13–15]. Previous studies using the simultaneous presentation of upright and inverted biological motion, as in the currently reported research, have shown that toddlers with ASD attended less to upright biological motion than did TD toddlers [13, 16], while preschool children with ASD showed stronger preference for upright biological motion, compared to TD preschool children [15]. One possible explanation is that developmental changes in orientation preference for biological motion might continue until adulthood. Comparison of percentage fixation times for upright vs. inverted biological motion, as assessed using Gazefinder, between preschool children [15] and adults (present study) reveals that TD preschool children show a preference for upright orientation over inverted orientation (51.6 % for upright and 34.5 % for inverted) [15], while TD adults show equivalent fixation times to upright and inverted biological motion in the current study (52.7 % for upright and 44.7 % for inverted). Similar to TD participants, children with ASD showed a stronger preference for upright orientation (55.5 % for upright and 27.4 % for inverted) [15], while adults with ASD did not show such a preference in this study (47.7 % for upright and 49.7 % for inverted). The findings suggest that preschool children’s preference for upright biological motion—when upright and inverted biological motion are presented simultaneously—might disappear after adolescence for both individuals with TD and ASD. With age, ASD participants’ preference—relative to upright orientation of biological motion—might become less prominent.

The percentage fixation times of the ASD group were lower for people and higher for geometry than those of the TD group for people and geometry (same size) and (small window). This result replicated previous studies with toddlers and children, which showed participants with ASD preferring to look at geometric images when geometric images and people are simultaneously presented [24, 25]. These findings suggested that the ASD group was consistently less likely to gaze at people in stimuli of this type, regardless of developmental stage.

### Relationship between percentage fixation times and social disability

For within-group correlation analysis, only two items, the mouth region of the faces in the (blinking) stimulus and the geometry region of the people and geometry (same size) stimulus, were correlated with SRS scores, and no item showed significant correlations in both the ASD and TD groups. The reason why almost no percentage fixation times to AoIs showed within-group correlations may be due to the different components that Gazefinder and SRS measure. SRS can measure a wide range of social deficits, while Gazefinder can only measure abnormality in eye contact, which is only one of the components in social deficits. Individuals with severe social deficit may show mild gaze abnormality, and vice versa. However, although use of a different scale (ADOS) for measuring social deficit needs to be taken into account, previous research on toddlers with ASD has found a significant correlation of social deficit with the fixation time to salient social information [5, 18]. Moreover, Nakano et al. [19] reported that children and adults with ASD have different gaze patterns. Thus, individuals with ASD may change their pattern of attention to social information with accumulating social experience, and of course, this is similar to individuals with TD. A reanalysis of the correlation between social deficit and abnormality in eye contact needs to be carried out with a longitudinal design.

However, within-group analysis showed significant correlations in the mouth region of faces in the (blinking) stimulus and in the geometry region of the people and geometry (same size) stimulus. For the mouth region, despite the fact that moving eyes normally attract attention, avoiding gazing to the eye region in this condition might reflect a severe social deficit. In addition, for the people and geometry (same size) stimulus, a previous study reported that 1.9 % of TD toddlers showed a preference for geometry, while 40 % of ASD toddlers did [24]. Thus, individuals with TD showed the strong preference for people in this stimulus, and the geometry region of the people and geometry (same size) may reflect the degree of social deficit sensitively in TD individuals. Therefore, the geometry region of this stimulus correlated with the SRS score significantly only in the TD group. These two items had some factors that reflect the degree of social deficit in each group and may enable measurement of the severity of the individual’s social deficit.

### Conclusion regarding group differences in percentage fixation times and relationship to social disability

Considering that IQ did not correlate with the percentage fixation times found with Gazefinder, we conclude that intellectual ability did not have a strong impact on the performance of Gazefinder. Therefore, we can state that Gazefinder could measure the unique patterns of gaze fixation to social information shown by adolescents and adults with ASD, using human face stimuli that omitted lip movement and using people and geometry stimuli, but at the same time, the unique gaze fixation patterns detected by Gazefinder did not sensitively predict the degree of social deficit in this age group. In a previous study using Gazefinder, we reported the unique fixation patterns of preschool children with ASD [15]. However, children and adults with ASD were reported to have a different gaze pattern [19]. Therefore, in order to use the Gazefinder successfully with an older group (i.e., adolescents and adults) with ASD, it was essential that we evaluate gaze patterns in individuals with ASD in this age group. In fact, we found the different gaze abnormalities revealed in our previous study; preschool children with ASD showed a gaze abnormality while viewing biological motion but not while viewing other stimuli [15]. Given these findings, our present study provided important evidence for the validity of using Gazefinder with the adolescent-to-adult participants with ASD.

### Discriminant analysis

When we used all the AoI-1s, sensitivity, specificity, PLR, and NLR were 61.9 %, 85.7 %, 4.3, and 0.4, respectively. However, when we selected the data on three AoI-1s that had exhibited large effect sizes (i.e., movies showing the eye region of the human face (blinking), the eye region of the human face (silent), and the people region of people and geometry (same size), the AUCs being .80, .75, and .74, respectively), we found that these were the more accurate discriminant parameters; sensitivity, specificity, PLR, and NLR were 81.0 %, 80.0 %, 4.1, and 0.2, respectively. Thus, we found better discriminant parameters with AoI-1s that showed large effect sizes on the percentage fixation times.

Were the discriminant parameters of Gazefinder at an acceptable level compared to other eye-tracking studies? Only one study with 4- to 6-year-old children by Shi et al. [25] reported AUCs and discriminant parameters [25]. Although the study design was not intended for clinical application and the participants’ age was different from that of the present study, Shi et al. reported that sensitivity and specificity were 84.6 and 85.0 %, respectively, using total fixation time on people, in data gathered while children viewed movies that simultaneously presented people and geometric stimuli [25]. When we used three items (eye region of the human face blinking) and (silent), and people region of people and geometry (same size)), we found sensitivity and specificity (i.e., above 80 %) that were comparable to that found by Shi et al. [25]. Therefore, we could conclude that the discriminant parameters of Gazefinder were at an acceptable level compared to a previous eye-tracking study, and its discriminant level was sufficient.

Are the discriminant parameters of Gazefinder sufficient compared to other tools that measure the ASD trait? The autism spectrum quotient (AQ), one of the most widely used self-administered questionnaires, discriminated adults with ASD from TD with a sensitivity of 79.3 % and a specificity of 97.7 % [38]. Compared to this, Gazefinder was equal to AQ for sensitivity, although the specificity of Gazefinder was relatively lower than that of AQ. Considering the easy and short administration (approximately 2 min) of Gazefinder, its discriminant parameters are sufficient relative to AQ, especially with regard to sensitivity.

### General discussion

Gazefinder has the advantages of ease of use, short testing time, passive viewing, and immediate calculation of objective results, which we conjectured would overcome the limitations of previously available assessment and diagnostic tools. For example, ADOS-2 and ADI-R, the most widely used diagnostic tools, demand a high level of expertise and a long time for administration. Moreover, individuals with ASD may have difficulty in expressing their ASD trait accurately on self-reported questionnaires [39]. Although we should bear in mind that Gazefinder can measure only a small part of ASD symptomatology, from the Gazefinder’s advantages described above, we conclude that Gazefinder may be useful as a supplementary clinical tool for objectively measuring unique gaze fixation patterns. It is important that anyone in the healing professions be able to assess gaze abnormality objectively and with the same criteria, and we believe that such an achievement will lead to increasing the validity of an ASD diagnosis all over the world. In addition to this, one participant with ASD in our research had difficulty completing the SRS, which underscores the fact that use of Gazefinder, in which participants simply look at a monitor, is especially helpful for diagnosis in such individuals.

Finally, we emphasize a few notes of caution for using Gazefinder in clinical contexts at this moment. Firstly, we should use Gazefinder only as a supplementary tool for ASD assessment and diagnosis, and clinicians should not make definitive ASD diagnoses solely based on it. Although high sensitivity and specificity were shown in our research, there were probabilities of false positives and negatives even in clearly defined ASD and TD groups. This could be because Gazefinder measures only one part of the wide range of ASD symptoms. Secondly, we need to understand the nature of the individuals to whom we try to apply Gazefinder. Because adult ASD is accompanied by other psychiatric disorders at high rates [40, 41], we may have few opportunities to carry out Gazefinder testing with adults with pure ASD in clinical settings; adults with unidentified ASD seen in specialized institutions have a strong probability of meeting the diagnostic criteria for some additional psychiatric disorder. Moreover, previous research has found that individuals with anxiety disorder [42, 43], schizophrenia [12, 44], and ADHD [45, 46] tend to gaze less at eye regions of the human face; a low percentage fixation to social information may emerge from other psychiatric disorders. Given this state of knowledge, clinical examinations of patients and interviews with caregivers remain necessary for diagnosis of comorbid disorders or for differential diagnosis. However, a previous study has reported that persons with schizophrenia oriented to social information more rapidly than persons with ASD [12], suggesting that we may be able to make definitive diagnoses of comorbid psychiatric disorders or discriminative diagnoses among psychiatric disorders with indexes that were not analyzed in this research. We think that achieving this is a task for the future. At present, we know only that Gazefinder detected patients with pure ASD who were sufficiently affected to visit a psychiatry department.

### Limitations and future directions

We note two limitations of our research. First, the sample size may not be sufficient. However, we found similar results in previous studies [3–9, 17–19, 24, 25], and there were significant group differences and large effect sizes in the gaze data from viewing movies of the human face that omitted lip movement and the movies of people and geometry. Therefore, we would predict the same outcomes even with a larger participant pool. Second, five participants in this study’s ASD group were excluded because of insufficient data on percentage fixation time (<80 %), and this high exclusion rate was of great concern. An investigation by JVC KENWOOD Corporation found that glass-wearing might be one reason for failures of eye tracking (percentage wearing glasses in the present study: TD, 35.3; ASD, 73.1). In addition, gazing with the head lowered, which is suggested to be more common in ASD, also might appear to be a factor that reduced the available percentage fixation times. However, Gazefinder has since been updated, with improved robustness against various ambient light conditions, glass lens reflection conditions, and subject head movements. In addition to this, we can assess whether Gazefinder detected participants’ fixations in real time. Therefore, we might well be able to decrease the participant exclusion rate by monitoring the system’s performance and attempting to capture the data again when it fails to obtain participant fixation data on the first attempt.

For the purposes of improving future research, we note two points. First, assessing the validity of Gazefinder in other groups with ASD is needed (e.g., different age groups and females). Confirmation of validity with diverse populations will be important for clinical use. Next, as described in the “Discussion” section, we should also examine the discriminant analysis of Gazefinder between ASD and other disorders. Revealing the qualitative and quantitative differences in gaze fixation between ASD and these disorders, for example anxiety disorder or schizophrenia, could be helpful for both differential diagnosis and refinement of the characterizations of these disorders.

## Conclusions

Our results indicated that Gazefinder might be an easy-to-use tool for quantitatively measuring unique gaze fixation patterns of ASD and for discriminating male adolescents and adults with ASD from TD participants in clinical contexts.

## Additional file

Additional file 1: A rationale for the design of Gazefinder.

## Abbreviations

ADOS: autism diagnostic observation schedule
AoI: area-of-interest
AQ: autism spectrum quotient
ASD: autism spectrum disorder
AUC: area under the curve
DISCO: Diagnostic Interview for Social and Communication Disorders
FSIQ: full-scale IQ
IQ: intelligent quotient
NLR: negative likelihood ratio
PLR: positive likelihood ratio
ROC: receiver operating characteristic
SRS: social responsiveness scale
TD: typically developed
WAIS-III: Wechsler Adult Intelligence Scale—Third Edition
WISC-III: Wechsler Intelligence Scale for Children—Third Edition
