# Supplementary material for: Gazefinder as a clinical supplementary tool for discriminating between autism spectrum disorder and typical development in male adolescents and adults
Source: Mol Autism. 2016 Mar 23;7:19. doi: 10.1186/s13229-016-0083-y (PMC4804639; doi:10.1186/s13229-016-0083-y)
Supplement: Additional file 1: — A rationale for the design of Gazefinder. [file 13229_2016_83_MOESM1_ESM.docx]

**Additional file 1**

**A rationale for the design of Gazefinder**

As noted in the manuscript, Gazefinder contained five face movies and two people-and-geometry movies. The rationale for having five face movies was as follows. From previous studies that examined various face conditions, we assumed that individuals with ASD might show different unique gaze patterns between the still-face condition and the social interaction condition [1, 2]. Therefore, we developed [still image] and [talking] stimuli. In addition to this, based on Chawarska et al. (2012), we assumed that toddlers with ASD gaze at moving objects per se [3]. Moreover, because the actress blinks and moves her mouth during the [talking] stimulus, we developed [blinking] and [mouth moving] stimuli to detect differences in gaze patterns between talking stimuli and stimuli only showing moving parts of faces. In addition, we provided a [silent] stimulus, which was shown between [mouth moving] and [talking] stimuli, for releasing the participant’s attention from the moving object.

We set the default duration of face stimuli at 7 s initially, because the duration of talking in the human face [talking] movie was this time span. However, considering results of our preliminary study, we needed to shorten the total presentation time of stimuli because of the limited attention spans of infants. Therefore, we decided to decrease the presentation time of [mouth moving], which one has little chance to see in real life, and [silent], which is unoccupied time used to release participants’ attention from moving objects.

The rationale for having two people-and-geometry movies was as follows. First, we employed the [same size] stimulus that was used in previous research [4, 5]. However, using the [same size] stimulus, Pierce et al. (2011) reported that the percentages of fixation to geometry of the ASD group were evenly distributed from 0% to 100%, while the percentages of fixation to geometry of the TD group were distributed mainly from 0% to 50%. Based on this, we hypothesized that “children with ASD visually monitor randomly and do not show any preference for geometry or people, while TD children show a preference for people.” For this reason, we assumed that if specific individuals with ASD showed a preference for geometry over people, the results would express the preference even if the area allocated to presentation of geometry were smaller than that of people, as in the [small window] movie. Therefore, we developed the movies of people-and-geometry [same size] and [small window].

**References**

1. Chawarska K, Macari S, Shic F. Decreased spontaneous attention to social scenes in 6-month-old infants later diagnosed with autism spectrum disorders. Biol Psychiatry. 2013;74(3):195-203. doi:10.1016/j.biopsych.2012.11.022.

2. Merin N, Young GS, Ozonoff S, Rogers SJ. Visual Fixation Patterns during Reciprocal Social Interaction Distinguish a Subgroup of 6-Month-Old Infants At-Risk for Autism from Comparison Infants. J Autism Dev Disord. 2007;37(1):108-21. doi:10.1007/s10803-006-0342-4.

3. Chawarska K, Macari S, Shic F. Context modulates attention to social scenes in toddlers with autism. J Child Psychol Psychiatry. 2012;53(8):903-13. doi:10.1111/j.1469-7610.2012.02538.x.

4. Pierce K, Conant D, Hazin R, Stoner R, Desmond J. Preference for Geometric Patterns Early in Life as a Risk Factor for Autism. Arch Gen Psychiat. 2011;68(1):101-9. doi:DOI 10.1001/archgenpsychiatry.2010.113.

5. Shi L, Zhou Y, Ou J, Gong J, Wang S, Cui X et al. Different visual preference patterns in response to simple and complex dynamic social stimuli in preschool-aged children with autism spectrum disorders. PLoS One. 2015;10(3):e0122280. doi:10.1371/journal.pone.0122280.
